# The Potential Role of Fish-Derived Protein Hydrolysates on Metabolic Health, Skeletal Muscle Mass and Function in Ageing

**DOI:** 10.3390/nu12082434

**Published:** 2020-08-13

**Authors:** Matthew J. Lees, Brian P. Carson

**Affiliations:** 1 Department of Physical Education and Sport Sciences, Faculty of Education and Health Sciences, University of Limerick, V94 T9PX Limerick, Ireland; matthew.lees@ul.ie; 2 Health Research Institute, University of Limerick, V94 T9PX Limerick, Ireland

**Keywords:** amino acids, sarcopenia, leucine, protein synthesis, appetite

## Abstract

Fish protein represents one of the most widely consumed dietary protein sources by humans. The processing of material from the fishing industry generates substantial unexploited waste products, many of which possess high biological value. Protein hydrolysates, such as fish protein hydrolysates (FPH), containing predominantly di- and tripeptides, are more readily absorbed than free amino acids and intact protein. Furthermore, in animal models, FPH have been shown to possess numerous beneficial properties for cardiovascular, neurological, intestinal, renal, and immune health. Ageing is associated with the loss of skeletal muscle mass and function, as well as increased oxidative stress, compromised vascularisation, neurological derangements, and immunosenescence. Thus, there appears to be a potential application for FPH in older persons as a high-quality protein source that may also confer additional health benefits. Despite this, there remains a dearth of information concerning the impact of FPH on health outcomes in humans. The limited evidence from human interventional trials suggests that FPH may hold promise for supporting optimal body composition and maintaining gut integrity. FPH also provide a high-quality source of dietary protein without negatively impacting on subjective appetite perceptions or regulatory hormones. Further studies are needed to assess the impact and utility of FPH on skeletal muscle health in older persons, ideally comparing FPH to ‘established’ protein sources or a non-bioactive, nitrogen-matched control. In particular, the effects of acute and chronic FPH consumption on post-exercise aminoacidaemia, skeletal muscle protein synthesis, and intramyocellular anabolic signalling in older adults are worthy of investigation. FPH may represent beneficial and sustainable alternative sources of high-quality protein to support skeletal muscle health and anabolism in ageing, without compromising appetite and subsequent energy intake.

## 1. Introduction

Fish protein represents one of the most widely consumed dietary protein sources by humans, with global fish production peaking at around 171 million tonnes in 2016 [1]. In particular, the Scandinavian countries consume substantial amounts of fish, with Sweden and Norway consuming approximately 50 kg and 30 kg per capita, respectively, as of 1996 [2]. The processing of material from the fishing industry generates more than 60% of its by-products as rest, or waste, comprising skin, head, viscera, trimmings, liver, frames, bones and roes [3]. These by-products represent valuable, high-quality sources of protein and nutrients that are underused and/or unexploited [4,5,6]. Enzymatic hydrolysis is the preferred method of extracting these resources in the food and pharmaceutical sectors, as other methods (i.e., chemical hydrolysis) can leave residual organic solvents or chemicals in the products [7]. Using this technique, protein biomass may be recovered from waste products that would otherwise be discarded [8], thus improving economic and environmental sufficiency/sustainability [9,10].

Protein hydrolysates providing mainly di- and tripeptides, the major protein fragments in fish protein hydrolysates (FPH), are absorbed more readily than free amino acids (AAs), and much more rapidly than intact protein [11,12]. The various FPH are breakdown products from the enzymatic conversion of fish proteins into smaller peptides, and normally contain 2–20 AAs [3]. In recent years, FPH have attracted considerable attention from food biotechnologists, given the abundance of raw material for the process, its high protein content, and desirable AA profile [3,12]. Importantly, this material is host to a plethora of bioactive peptides that may have practical applications in functional foods [3,5,8,10,13,14,15].

In vitro studies have suggested the beneficial properties of FPH-derived bioactive peptides, with potential applications for intestinal health [16,17], serum and liver cholesterol/lipid profile [18,19,20,21], fatty acid metabolism [22], postprandial glucose control [21], and hypertension [23]. There is also evidence in rodents that fish protein may increase plasma AA concentrations, their transport pathways [24], and associated fast-twitch muscle gene expression and mass [20]. Bioactive peptides released in the process of hydrolysation may also possess desirable immunomodulatory, anti-inflammatory, antioxidant, and neuroprotective effects [25,26,27,28]. These beneficial properties have potential implications for older people, given that chronic inflammation [29], neurological derangements [30], compromised vascularisation [31], increased oxidative stress [32], alterations in the gut microbiome [33], impaired glucose homeostasis [34], and immunosenescence [35] are all linked with advancing age.

In ageing, the progressive loss of muscle mass, strength, quality, and function, otherwise referred to as sarcopenia [36], is associated with a range of adverse health outcomes in humans. These include, but are not limited to, functional impairment [37,38], physical disability [39], decreased quality of life [40], an increased risk of falls [41], hospitalisation [42], and mortality [43]. The provision of dietary protein, particularly when consumed alongside resistance exercise, is recognised as an important strategy to manage and prevent sarcopenia in older persons [44,45,46,47]. Despite this, 31–50% of non-institutionalised older adults fail to meet the current Recommended Daily Allowance (RDA) of 0.8 g·kg^−1^·body mass^−1^·day^−1^ [48]. Moreover, several authors have advocated protein intakes in excess of 1.0 g·kg^−1^·body mass^−1^·day^−1^ for this population [49,50,51,52]. Although older people may be less responsive to low-dose AA servings, this ‘anabolic resistance’ may be overcome by optimising protein or essential amino acid (EAA) provision [49,53,54] and resistance exercise [46]. Somewhat paradoxically, it is well established that dietary protein has a dose-dependent satiating effect [55,56] that has implications for older people with dysregulated appetite perception and/or appetite hormones [57,58]. Therefore, efficacious protein formulations are required to address nutritional deficiencies in older people without compromising appetite and energy intake. FPH might represent a potential means of meeting this need.

To date, one review has summarised the existing knowledge from animal and human clinical studies evaluating the effects of lean fish, fish proteins, and fish-derived peptides on metabolic health outcomes [5]. However, to our knowledge, the current evidence relating to FPH within the context of adult human health and ageing has not been outlined. Therefore, the purposes of this review were to (1) explore the present evidence on the impact of fish-derived protein formulations in adult human interventional studies, and (2) investigate the current and potential applications of these formulations on skeletal muscle health and function in ageing.

## 2. Literature Search

We searched the US National Library of Medicine (PubMed) and MEDLINE databases for human interventional studies, using ‘fish protein’, ‘marine protein’, ‘fish protein hydrolysate’, ‘marine protein hydrolysate’ and ‘human’ as key terms. The Boolean operators “AND” and “OR” were used to combine search terms. Additional literature was obtained from ‘related articles’ searches and by manually screening the reference lists of included studies. The literature search identified 349 articles that were subsequently assessed for duplicate entries. Following screening for non-human studies, those that did not investigate FPH, and studies in paediatric populations, a total of 14 articles were included for review (Table 1). Only refereed articles published in English language peer-reviewed journals from January 1980 until May 2020 were included. Studies published in abstract form or in the grey literature were not considered.

## 3. Regulation of Human Physiological Function by Fish Protein Hydrolysates

An overview of studies investigating the effects of FPH on human health is provided in Table 1.

### 3.1. Vascular Function

In a rodent model, FPH was shown to decrease systolic blood pressure and exert an inhibitory effect on angiotensin-I-converting enzyme [23]. In healthy humans, two studies explored the impact of FPH formulations on vascular function in the same laboratory, using similar designs [59,60]. Both investigations provided single FPH (hydrolysed from Nile tilapia) doses of either 5 g [59] or 20 g [60] and monitored vascular responsiveness at the brachial artery using flow-mediated dilation (FMD). Furthermore, both studies also measured the total antioxidant capacity (TAC) of each supplement using the Trolox equivalent antioxidant capacity assay. Oliveira et al. [60] found no beneficial effect of FPH over placebo (PLA) or whey protein hydrolysate (WPH) on FMD at baseline or during 120 min post-supplementation. This was in spite of the fact that the TAC of the FPH supplement was greater than PLA (239.17 ± 3.91 versus 25.56 ± 0.00 mM Trolox Equivalent (TE)/20 g fresh weight; *p* ≤ 0.001) and WPH (40.09 ± 13.32 mM TE/20 g fresh weight; *p* ≤ 0.001), respectively. The TAC of the WPH supplement was comparable to that of the PLA (*p* = 0.205). Consistent with Oliveira et al., Alvares et al. [59] found no beneficial effect of FPH over PLA on FMD values during 120 min post-supplementation. In addition, microvascular function was measured using near-infrared spectroscopy (NIRS) to quantify tissue O_2_ saturation (StO_2_) in response to a vascular occlusion test at the brachial artery. No significant interaction effects were found between supplements for NIRS-derived StO_2_. In agreement with Oliveira et al., the TAC of the FPH supplement was greater compared with PLA (569.4 ± 75 versus 0.0 mM TE/100 g; *p* < 0.05). Both studies recruited nine participants, with Alvares and colleagues acknowledging the low statistical power to detect differences between FPH and PLA (1 − β error = 0.08 − 0.37).

Although the beneficial anti-hypertensive and antioxidant properties of FPH have been demonstrated in vitro [23,28,61] the existing evidence in humans suggests no benefit of FPH on arterial vascular responsiveness, despite its significantly greater TAC [59,60]. Indeed, the demonstration of bioactive properties in vitro does not necessarily infer that the same beneficial effects will be observed following human consumption [62]. It must also be recognised that the two human studies discussed here did not establish the in vivo postprandial redox state following supplementation, which would have been informative. The effects of FPH on vascular function and its mechanistic underpinnings in longitudinal studies and/or populations with elevated cardiovascular risk factors also remain to be elucidated. Such findings may be of benefit to older persons, as compromised vascular responsiveness has been posited to impede nutrient delivery (i.e., AAs) to skeletal muscle in this population [31], however the evidence for this is controversial. The nutraceutical and pharmaceutical modulation of skeletal muscle blood flow has not been shown to enhance muscle protein synthesis (MPS) in the presence of dietary protein or EAAs in younger and older adults [63,64,65,66]. Contrastingly, others have observed a positive effect of increased skeletal muscle perfusion and AA provision on MPS in older adults [67]. The purported beneficial impact of FPH on skeletal muscle blood flow, nutrient delivery, and MPS therefore remains to be established in humans.

### 3.2. Appetite Regulation

Four studies were found that investigated the impact of FPH on subjective appetite ratings and/or circulating appetite hormones in healthy adults [69,70,76,79]. The earliest investigation by Zaïr et al. [79] found no significant differences between a blue whiting-derived FPH and PLA on area under the curve (AUC) subjective hunger perceptions or biomarkers of appetite/satiety including plasma cholecystokinin (CCK) and glucagon-like peptide 1 (GLP-1). Similarly, no difference in ad libitum energy intake was found between conditions. Nobile et al. [76] found that total fasting plasma CCK and GLP-1 concentrations were increased following 90 days of blue whiting-derived FPH supplementation (1.4 g versus 2.8 g per day) and to a greater extent compared with PLA. Both CCK and GLP-1 are insulinotropic peptides known to stimulate satiety [80,81]; however, complementary subjective ratings of appetite (i.e., visual analogue scales [82]) in this study were not measured. More recent work by Dale et al. [70] found no differences in GLP-1 concentrations after consuming 20 mg·kg^−1^·body mass^−1^ of either an FPH derived from Atlantic cod or a casein control. In a similar study using the same cohort, postprandial and total AUC concentrations of acylated ghrelin, a peripheral hormone with orexigenic properties, did not differ between FPH and control [70]. Similarly, no between-group differences were found for subjective satiety scores and feelings of fullness. Lastly, Jensen and colleagues [73] conducted a dose-ranging crossover study (daily supplementation for one week) of a cod-derived FPH in healthy older volunteers aged 60–78 years. No differences in the estimated maximum value or AUC of serum GLP-1 were observed when comparing the lowest dose of FPH (10 mg·kg^−1^·body mass^−1^) against the higher doses (20, 30, or 40 mg·kg^−1^·body mass^−1^), suggesting the FPH was ineffective. The lack of a placebo control further complicates the interpretation and significance of these findings.

Taken together, these findings suggest that—irrespective of source species—the consumption of FPH does not detrimentally impact appetite regulation or subsequent ad libitum energy intake in healthy young and middle-aged participants. However, to date, no studies have been undertaken on the role of FPH in appetite regulation, its potential impact on protein intake, and subsequent effects on MPS and skeletal muscle health in older adults.

### 3.3. Glycaemic Control

Four studies incorporated measures of blood glucose and/or insulin concentrations in response to FPH consumption. Zaïr et al. [79] explored the impact of two separate doses of FPH compared with PLA over the course of a 420 min acute trial in overweight but otherwise healthy, normoglycaemic women. Trials were preceded by two weeks of supplementation with two daily doses of 1 g FPH or PLA. Plasma glucose was significantly lower at 270 min post-consumption of the first 333 mg dose (i.e., 30 min following the consumption of the second identical dose) in the FPH condition compared with PLA (4.71 ± 0.42 versus 4.93 ± 0.51 mmol·L^−1^; *p* < 0.05). Interestingly, there were no significant differences in plasma insulin concentrations between groups at any measurement point. Although the omega-3 polyunsaturated fatty acid content of the supplement was not specified, eicosapentaenoic acid (EPA) has been shown to mediate skeletal muscle glucose uptake in murine C2C12 cells, through AMP-activated protein kinase (AMPK) signalling and glucose transporter 4 (GLUT4) translocation [83]. In addition, omega-3 index (i.e., red blood cell concentrations of EPA and docosahexaenoic acid (DHA)) was positively associated with insulin sensitivity (*r* = 0.23; *p* = 0.025) in a study of 47 overweight, middle-aged males [84].

In contrast with Zaïr et al., Dale et al. [69] found no differences in serum glucose between FPH and a casein control for either time series, peak concentration, or postprandial AUC. In a mixed-model linear regression analysis, serum insulin concentration was significantly lower after FPH consumption compared with the control (mean difference between geometric means (95% confidence interval (CI)): 1.067 (1.01, 1.13) mIU·L^−1^; *p* = 0.032). However, no significant differences in peak concentration or AUC values were reported. The reduced insulin concentration in the absence of changes in blood glucose in this study run counter to the findings of Zaïr et al., potentially due to the choice of FPH (blue whiting versus cod) and/or the use of a two-week supplementation period by Zaïr et al. However, without more detailed information on the supplement composition in Zaïr et al., it is difficult to substantiate these notions. Taken together, these findings suggest that FPH may have a role in the regulation of glucose uptake and insulin sensitivity in young adults.

In older volunteers, Jensen et al. [73] performed a dose-ranging crossover study that found no difference in the estimated maximum value of serum glucose or insulin when comparing the lowest dose of FPH against the higher doses. Furthermore, no differences were found in AUC for these measures when comparing the lowest dose with the higher doses. However, the lack of a dose–response is difficult to interpret without the context of a placebo control. It should also be noted that the doses of FPH used in this study were considerably lower (40 mg·kg^−1^·body mass^−1^ at the upper limit) when compared with other studies of FPH and/or WPH [68,85]. Although serum glucose and insulin appeared to decline with increasing amounts of FPH, these changes did not attain statistical significance. These data suggest that higher doses of FPH may be beneficial for glucose control, but this requires further substantiation.

In a feasibility study of older (>80 years) nursing home residents, Drotningsvik et al. [72] investigated the impact of six weeks daily supplementation with 5.2 g of blue whiting-derived FPH versus a placebo on serum markers of glucose metabolism (i.e., fasting glucose, insulin, and fructosamine). Participant completion was high (88%), and exceeded that reported in previous supplementation studies in older persons [86,87]. Although the study was feasible and safe, with a low dropout (*n* = 3), recruitment was difficult given the target population. In addition, the intervention did not affect fasting serum glucose, insulin, and fructosamine.

Collectively, these findings suggest that in young and older volunteers, there is some evidence for a benefit of FPH supplementation on glycaemic control, but much further work is required in this area. The inconsistent findings from the aforementioned studies mirror the broader literature, particularly with regard to moderate (i.e., 2–3 g) and high dose (>5 g) omega-3 polyunsaturated fatty acid supplementation [88]. Although the administration of FPH appears to be safe and does not pose deleterious consequences for glucose homeostasis, more high-quality randomised controlled trials are necessary to reliably determine the impact of FPH on insulin sensitivity and glycaemic control. Such investigations may measure postprandial glucose and insulin concentrations following an oral glucose tolerance test, or the gold-standard hyperinsulinaemic–euglycaemic clamp technique [89]. The findings from these trials may be of particular importance for older persons, given that glycaemic control may be impaired in this population due to losses in skeletal muscle mass and function that predispose individuals to reduced skeletal muscle insulin sensitivity [34,90].

### 3.4. Body Composition

Nobile et al. [76] found that 90 days of FPH supplementation (1.4 g or 2.8 g) combined with a daily caloric deficit of 300 kcal decreased body weight, body mass index, dual-energy X-ray absorptiometry (DXA)-derived fat mass, and waist, hip, and thigh circumference to a similar extent compared with a whey protein isolate control. No significant changes were observed in extracellular water, and there were no significant differences between FPH doses or the whey protein isolate control for all measured endpoints. Measures of lean mass, fat-free mass, and bone mineral content by DXA were not reported; hence, the contributions of these tissues to the overall body weight reductions, as per the three-compartment model [91], are unclear. From these data, it is inconclusive that the FPH had any impact on body composition as the observed changes are likely attributable to the caloric deficit achieved through alimentary diaries.

### 3.5. Plasma Aminoacidaemia

The availability of AAs is crucial for the regulation of MPS [92], with rapid aminoacidaemia serving to enhance skeletal muscle anabolism and MPS following resistance exercise [93]. Dietary protein, particularly EAAs, drives anabolism and MPS through mammalian target of rapamycin complex 1 (mTORC1) signalling [44,94,95]. Among the EAAs, leucine is well known for its unique ability to stimulate mRNA translation via mTOR signalling and that of its downstream effectors ribosomal protein S6 kinase (p70S6K), eukaryotic translation initiation factor 4E-binding protein 1 (4E-BP1) and ribosomal protein S6 kinase (rpS6) [94,96]. Hence, the leucine and EAA content of a given protein source are key considerations for skeletal MPS.

Of the 14 studies included in this review, the AA composition of each FPH (if available) is provided in Table 2. There was considerable variation in the AA profile of each FPH, both within and between species, as well as among the WPH comparators.

One study explored the impact of a Nile tilapia-derived FPH compared with WPH on post-exercise aminoacidaemia in healthy young individuals [68]. Immediately following a resistance exercise bout, 0.25 g·kg^−1^·body mass^−1^ of oral FPH, WPH, or PLA were consumed. Significant increases in total AAs, EAAs, branched-chain amino acids, and leucine were observed in the FPH trial at 30 and 60 min postprandial compared with PLA. There were no significant differences between FPH and WPH at any time point or for AUC concentrations. This is surprising, given the reduced leucine and EAA content compared with WPH. The effects of FPH formulations from other species and/or those providing greater amounts of leucine on plasma aminoacidaemia and skeletal muscle anabolism remain to be determined. These findings indicate that this particular FPH formulation induced comparable plasma aminoacidaemia to a WPH formulation and thus may represent a viable alternative source of high-quality dietary protein for supporting the maintenance of skeletal muscle mass.

### 3.6. Intestinal Health

Non-steroidal anti-inflammatory drugs (NSAIDs) are widely used by older persons, with 7.3% of all individuals over 65 years of age filling at least one prescription for reimbursed NSAIDs during a 12-month period [98]. An increase in gastrointestinal (GI) tract permeability may result in the decreased absorption of nutrients, particularly AAs, that play a crucial role in skeletal muscle health [92,99].

The impact of a pacific whiting-derived FPH compared with PLA on gut permeability was investigated by Marchbank et al. [74]. Participants consumed 1 g capsules of FPH or PLA three times daily for a total of seven days. After two days of supplementation, the NSAID indomethacin, a substance known to negatively affect the integrity of the small intestine, was added (50 mg three times daily for five days). Changes in gut permeability were measured using 5 h urinary lactulose/rhamnose (L/R) ratios. In the absence of indomethacin, FPH did not affect gut permeability. In response to indomethacin during the PLA arm, gut permeability increased by approximately five-fold. However, in the FPH arm, the increase in gut permeability was truncated, suggesting a protective effect against indomethacin-induced injury [74]. Dyspepsia was present in 50% of participants in the PLA arm, but none in the FPH arm. Although ageing itself can increase the risk of bleeding in the GI tract, GI ulceration and bleeding can be exacerbated in older persons through NSAID use [100]. This process is driven by the NSAID-induced inhibition of prostaglandin synthesis, causing a weakening of the mucosal barrier that protects the GI tract [100]. These findings indicate that short-term supplementation with a pacific whiting-derived FPH may be beneficial for preventing NSAID-induced intestinal injury, but the evidence for chronic supplementation is limited at present.

### 3.7. Inflammation

Two studies investigated markers of inflammation in response to FPH consumption. In the chronic study by Drotningsvik et al. [72] in nursing home residents, serum concentrations of the inflammatory marker monocyte chemoattractant protein-1 (MCP-1) were significantly reduced (mean difference (95% CI): −80 (−146, −13) pg·mL^−1^; *p* = 0.0064) after six weeks of supplementation in the FPH group, whereas C-reactive protein (CRP) significantly increased compared with the control group (0.77 (−6.83, 8.37) mg·L^−1^; *p* = 0.040).

Dale et al. [71] examined the effects of daily supplementation with cod-derived FPH (2.5 g) versus a placebo for six months on serum concentrations of gut integrity markers (intestinal fatty acid binding protein (iFABP), lipopolysaccharide-binding protein (LBP), and zonulin) and pro-inflammatory cytokines (tumor necrosis factor alpha, interferon gamma, interleukin 4, 6, 8, and 10) in irritable bowel syndrome (IBS) patients. Although there was no significant change in these markers in either group, the impact of larger doses of FPH and/or FPH derived from other species on these aspects remains to be established.

Hence, given these inconclusive findings, further investigation is necessary to explore the impact of FPH on inflammatory markers and the associated impact on skeletal muscle health and function in older adults.

### 3.8. Skeletal Muscle Function and Exercise Performance

Four studies were found that investigated the effects of FPH on exercise performance and physical function in trained individuals and older nursing home residents, respectively.

In moderate to well-trained cyclists, Vegge, Rønnestad, and Ellefsen [78] assessed 5-min mean-power performance following 120 min of steady-state cycling at 50% maximal aerobic power after the consumption of three beverages: carbohydrate alone (CHO; 8.3% maltodextrin; 60 g·h^−1^), PROCHO (CHO plus 2.1% intact whey protein; 15.3 g·h^−1^), and NpPROCHO (PROCHO plus 0.4% Nutripeptin^TM^ (Np); 2.7 g·h^−1^). No differences in 5-min mean-power performance were observed between the three beverages. However, an ergogenic effect on this measure was noted in the six lesser performing participants compared with the six better performing participants in NpPROCHO relative to ingestion of CHO alone. This preliminary evidence suggests a potential ergogenic benefit when supplementing with FPH in athletes at a lower level of performance.

In healthy aerobically trained males, 5-km time trial (TT) time to completion and power output did not differ when 2.4 g of FPH were added to a CHO-PRO beverage (53.1 g·h^−1^ maltodextrin and 13.6 g·h^−1^ whey protein concentrate) versus CHO-PRO and CHO alone [77]. Respiratory exchange ratio (RER) during a steady-state phase (50% of maximal power output for 90 min) was higher in CHO-PRO compared with CHO alone and CHO-PRO supplemented with FPH, suggesting greater fat oxidation in the latter two beverages. Therefore, although the FPH condition appeared to influence metabolism during the steady-state phase, these effects did not translate into a beneficial performance outcome.

Contrastingly, Mjøs et al. [75] explored the impact of FPH on short-term recovery between cycling bouts in healthy middle-aged cyclists. They found no difference in time to exhaustion, heart rate, RER, blood lactate, and glucose following the addition of FPH (20 mg) to a whey protein and carbohydrate formulation compared with an isoenergetic, isonitrogenous FPH-free supplement. The small dose of FPH may not have been sufficient to exert a substantial effect, and/or it may have been confounded by the simultaneous provision of whey protein and carbohydrate (12% and 66% of the energy composition from each beverage, respectively). Moreover, the doses of FPH used in all three exercise performance studies were small, and the influence of larger doses, acutely or chronically, on fat oxidation rates in cycling are not yet established. At present, these findings appear to reinforce the notion that for highly trained athletes, carbohydrate availability is the rate-limiting factor for performance in events lasting up to 3 h [101]. Therefore, the performance data presented here do not substantiate the use of FPH in this context.

Lastly, in the feasibility study of older (>80 years) nursing home residents by Drotningsvik et al. [72], body weight and handgrip strength were similar between groups at baseline and did not significantly change by the endpoint (data not presented). The amount of protein provided here (5.2 g per day) and the duration of supplementation may have been insufficient to attain a beneficial effect in this population. For example, Björkman et al. [102] observed a 2.1% increase in body weight after six months of supplementation with whey protein (20 g·day^−1^) in nursing home residents, whereas body weight declined by 1.9% in a control group. Although handgrip strength responses were similar between groups, individuals in the whey protein group required less physical assistance after the trial period. It is also important to note the synergistic role of resistance exercise alongside dietary protein for driving improvements in skeletal muscle mass and strength [45,46]. Frail older adults have been shown to respond to 24 weeks of dietary protein supplementation (30 g·day^−1^) when engaging in a progressive resistance exercise programme [103]. Lean mass increased by approximately 1.3 kg in the protein group and did not change in a placebo group, whereas handgrip strength increased in both groups. These findings highlight the role of protein intake in supporting skeletal muscle mass gains in frail older adults whilst underlining the integral value of resistance exercise training for strength and functional performance. The utility of FPH supplementation when allied to a programme of resistance exercise training remains to be explored in older and/or frail populations.

Taken together, these findings do not yet support the use of FPH in the context of the performance and functional outcomes measured in the above studies.

## 4. A Potential Role for FPH in Maintaining Skeletal Muscle Health and Preventing Age-Associated Sarcopenia

To date, there is a dearth of human studies investigating the bioactivity of FPH, and as such, there is great opportunity for future research to build on preliminary work in this area. Despite the limited evidence available at this time, the findings of this review have highlighted potential beneficial outcomes related to skeletal muscle health and mass through supplementation with FPH. At present, only one registered clinical trial is investigating the utility of FPH on measures of skeletal muscle health and function in older adults [104], thus further illustrating this area of opportunity.

Dietary protein is recognised as a key nutrient for older persons, particularly in relation to skeletal muscle mass, function, and health [99,105]. Numerous authors have advised intakes in excess of the present RDA for this population [49,51,52,105,106]. These recommendations are supported in part by the reduced anabolic sensitivity of skeletal muscle in older adults that may be brought about by any of a number of factors: reduced physical activity levels [107], increased splanchnic AA retention (particularly leucine) leading to attenuated aminoacidaemia [108], low-grade inflammation [109,110], reduced skeletal muscle amino acid transporter expression [111,112], and blunted anabolic signalling [51,113]. However, these dietary protein recommendations may pose a challenge for older individuals with compromised appetite and/or dysregulated appetite hormones [57], given that dietary proteins have a dose-dependent satiating effect [52,55,56,114]. The absence of deleterious effects on subjective appetite perceptions and regulatory hormones following FPH consumption in young and middle-aged participants [69,70,76,79] is promising but requires verification in older persons. To date, no study has explored the effects of FPH on appetite regulation in older individuals.

Irrespective of the daily protein requirement, the widespread use of NSAIDs in older persons and the increased susceptibility to GI tract ulceration and bleeding may negatively affect nutrient (and particularly AA) delivery, thus posing consequences for skeletal muscle health. The protective role of FPH on NSAID-induced injury in younger volunteers was demonstrated by Marchbank et al. [74]; however, this effect remains to be corroborated in older populations. FPH may hold promise as a useful prophylactic against NSAID-induced GI tract damage in older adults whilst also providing a high-quality source of dietary protein.

The compromised vascularisation associated with advancing age is proposed to hinder nutrient delivery to skeletal muscle [31], yet pharmaceutical and nutraceutical modulation of skeletal muscle blood flow has not reliably been shown to enhance MPS in the presence of dietary protein and EAAs in younger and older adults [63,64,65,66]. Despite promising findings in rodent models, there were no beneficial effects on vascular function in the two human studies reviewed here. This may have been a consequence of the low dose provided, or the small sample sizes recruited. To date, no study has investigated the effects of FPH on arterial vascular responsiveness in older volunteers. The greater TAC of the FPH beverage over PLA and WPH in both studies may influence vascular function and muscle health in older adults and warrants further exploration.

The comparable post-exercise aminoacidaemia observed in young individuals following FPH and WPH ingestion [68] indicates a potential role for FPH as an alternative and sustainable dietary protein source in ageing. Such FPH formulations may represent useful adjuncts to suboptimal protein sources [115] given the beneficial properties outlined above. Furthermore, FPH may also have applications in frail, prefrail, or under/malnourished older individuals [116]. The activation of MPS is driven to a substantial extent by the rapid postprandial increase in plasma leucine [93,94], and this effect is augmented by the provision of all EAAs, particularly following resistance exercise [117,118]. In skeletal muscle, mTORC1 is activated by leucine, and it positively promotes protein synthesis by phosphorylating two downstream effectors: p70S6K and 4E-BP1 [119,120,121]. After adjusting for participants’ body weight, the Nile tilapia-derived FPH utilised by Cordeiro et al. [68] contained less EAAs (approximately 24%) and leucine (approximately 31%) than WPH but nevertheless induced comparable postprandial aminoacidaemia. Although increased rates of MPS may seem to intertwine with translational signalling responses, the activation of mTORC1-associated substrates may persist long after MPS rates have normalised [122]. Therefore, future studies in older persons should integrate measures of protein synthesis to complement anabolic signalling targets in skeletal muscle. To our knowledge, no study has yet investigated the effects of FPH on anabolic signalling and MPS using in vitro, animal, or human models.

Lastly, future studies should seek to compare FPH formulations with ‘established’ bioactive sources, such as WPH, as well as non-bioactive nitrogen-matched controls [123], to more thoroughly comprehend the true effect of hydrolysed fish protein supplementation on outcomes related to muscle health and function. The reviewed studies explored the effects of FPH on various aspects of human physiology and its regulation, yet often these investigations used protein-free placebos (i.e., cellulose, sucralose, maltodextrin) or did not control for nitrogen content between conditions. Hence, future studies would benefit from more robust control of these variables to confidently reveal the utility of FPH supplementation for human ageing health.

## 5. Conclusions

At present, there is limited work on the in vivo bioactivity of FPH formulations in humans, despite promising research in animal models. There is also a paucity of literature concerning the impact of FPH on general health and skeletal muscle anabolism in older adults. Although fish-derived protein formulations appear to hold promise for promoting gut health, supporting body weight and fat mass losses, and driving post-exercise aminoacidaemia, these findings need to be corroborated in older populations. Further, comparisons of FPH with ‘established’ protein sources and/or non-bioactive protein controls would more cogently unravel the benefits of FPH. The potential applications of FPH as an alternative to whey-based formulations for older adults—particularly given the favourable subjective appetite ratings and regulatory hormone data—warrant further investigation. Future work should strive to understand the impact of fish-derived protein formulations on MPS and anabolic signalling responses, alone or in conjunction with resistance exercise.

## Figures and Tables

**Table 1:** Intervention trials exploring the effects of fish-derived protein hydrolysates on health, exercise performance, and ageing in healthy humans.

Reference	Participants	Research Design	FPH Source Species	Dosage	Exercise Protocol	Main Outcomes	**Key Findings**
Alvares et al. [59]	Healthy adults (6 males, 3 females; mean age not reported)	Randomised, double-blind, placebo-controlled, crossover trial	Nile tilapia—*Oreochromis niloticus*	5 g FPH or PLA (sucralose) dissolved in 100 mL water	N/A	*Primary outcomes*: Flow-mediated dilation (FMD), tissue oxygen saturation (StO2) *Secondary outcome*: total antioxidant capacity (TAC) of each beverage	FMD and StO2 parameters did not significantly change between FPH and PLA supplementation. TAC was significantly greater in the FPH supplement compared with the PLA.
Cordeiro et al. [68]	Physically active subjects (6 males, 3 females; age 27 ± 2 years)	Randomised, double-blind, placebo-controlled, crossover trial	Nile tilapia—*Oreochromis niloticus*	0.25 g·kg^−1^·body mass^−1^ WPH, FPH or PLA immediately following RE	3 sets × 10–12 RM leg press and leg extension	*Primary outcomes*: Plasma TAAs, EAAs, BCAAs, leucine (0, 30, 60, 90, 120, 180 min post-supplement)	Rapid and pronounced aminoacidaemia was observed following FPH and WPH, with no significant differences between protein sources at any time point.
Dale et al. [69]	Healthy, active individuals (15 males, 26 females; age 51 ± 6 years)	Randomised, double-blind, crossover trial	Atlantic cod—*Gadus morhua*	20 mg FPH or casein (control) per kg body weight before standardised breakfast	N/A	*Primary outcomes*: Serum glucose and insulin at 20 min intervals from 0 to 180 min postprandial *Secondary outcomes*: Plasma GLP-1 at the same time points	Postprandial serum insulin secretion was significantly lower following FPH, with no difference observed between FPH and control for serum glucose or plasma GLP-1.
Dale et al. [70]	Healthy individuals (15 males, 26 females; age 51 ± 6 years)	Randomised, double-blind, crossover trial	Atlantic cod—*Gadus morhua*	20 mg FPH or casein (control) per kg body weight before standardised breakfast	N/A	*Primary outcomes*: Plasma acylated ghrelin at 0, 20, 40, 80, 180 min postprandial *Secondary outcomes*: Subjective appetite sensations at the same time points	No effect of single-dose FPH on plasma acylated ghrelin, subjective satiety, or fullness sensations.
Dale et al. [71]	Irritable bowel syndrome (IBS) patients: FPH (*n* = 13, age 42.7 ± 11.9 years) or PLA (*n* = 15, age 45.1 ± 14.8 years)	Randomised, double-blind, placebo-controlled, parallel-intervention trial	Atlantic cod—*Gadus morhua*.	2.5 g FPH or PLA (maltodextrin) consumed daily for six weeks	N/A	*Primary outcomes:* IBS-SSS and QoL scores *Secondary outcomes*: Serum pro-inflammatory cytokines and gut integrity markers, faecal SCFAs and calprotectin	Total IBS-SSS scores reduced in both groups and did not differ at the end of the six weeks. No significant changes in serum pro-inflammatory cytokines, gut integrity markers or faecal measures.
Drotningsvik et al. [72]	Nursing home residents (intervention group: *n* = 12, age 84 ± 8 years; control group: *n* = 9, age 87 ± 5 years)	Randomised, double-blind, placebo-controlled pilot trial	Blue whiting—*Micromesistius poutassou*	5.2 g FPH or PLA daily for six weeks	N/A	*Primary outcome*: Feasibility of FPH intervention in older nursing home residents (participant compliance). *Secondary outcomes*: Serum markers of glucose metabolism and inflammation (MCP-1, CRP)	FPH intervention was feasible (completion rate = 88%). No significant between-group differences in baseline parameters. Serum MCP-1 was reduced and CRP increased from baseline to endpoint in the intervention group compared with the control group. Glucose, insulin, and fructosamine were unaffected by the intervention.
Jensen et al. [73]	Healthy older adults (13 male, 18 female; age 67.8 ± 4.9 years)	Randomised, double-blind, dose range crossover trial	Atlantic cod—*Gadus morhua*	10, 20, 30, or 40 mg·kg^−1^·body weight daily for one week with washout periods (one week) in between	N/A	*Primary outcomes*: Serum glucose and insulin at 20 min intervals for 2 h following FPH consumption and standardised breakfast on day 7 of each cycle. *Secondary outcomes*: Plasma GLP-1 at the same time points	No differences in estimated maximum value of insulin, glucose, or GLP-1 were found when comparing the lowest dose to higher doses. No significant differences in total AUC for any variable irrespective of dose.
Marchbank et al. [74]	Healthy volunteers (age 25–40 years; 5 males, 5 females)	Randomised, double-blind, placebo-controlled crossover trial	Pacific whiting	1 g FPH or placebo capsule three times daily for seven days followed by a two-week washout period, then the corresponding arm. Indomethacin (50 mg three times daily) was added for the final five days.	N/A	*Primary outcomes*: Gut permeability measured using 5 h urinary lactulose:rhamnose ratios. *Secondary outcome*: Dyspepsia incidence	Gut permeability increased approximately five-fold in placebo and indomethacin, but this effect was mitigated in the FPH arm. Dyspepsia was present in 50% of participants in the placebo arm but 0% in the FPH arm.
Mjøs et al. [75]	Healthy, trained, male cyclists (age 45.6 ± 5.3 years)	Randomised, double-blind, placebo-controlled trial	Atlantic cod—*Gadus morhua*	CHO-WP (PLA) or CHO-WP-FPH (20 mg FPH; isonitrogenous and isoenergetic beverages) following a cycling session, then 4 h recovery before an identical cycling session	20 min moderate intensity cycling at 60% VO_2_max; 90% VO_2_max for 5 min, then TTE at 95% VO_2_max	*Primary outcome*: TTE. *Secondary outcomes*: HR, RER, blood lactate, and glucose	No significant difference between supplementations measured by TTE, HR, RER, blood glucose, or lactate.
Nobile et al. [76]	Healthy, slightly overweight (25 kg/m^2^ ≤ BMI ≤ 30 kg/m^2^) male (25%) and female (75%) subjects (age approximately 40 years)	Randomised, double-blind, placebo-controlled, parallel trial	Blue whiting—*Micromesistius poutassou*	Either 1.4 g FPH, 2.8 g FPH, or whey protein isolate (PLA) daily for 90 consecutive days	N/A	*Primary outcomes*: Body composition (weight, fat mass, extracellular water, circumference of the waist, hips, and thighs) *Secondary outcomes*: Fasting plasma concentrations of total CCK and GLP-1	Treated subjects had improved body composition after 90 days. Measured end points did not differ significantly between 1.4 g and 2.8 g FPH. Increased fasting concentrations of CCK and GLP-1 were observed after 90 days in all conditions.
Oliveira et al. [60]	Healthy young (6 males, 3 females; age 22.5 ± 3.3 years), physically active adults	Randomised, double-blind, placebo-controlled, crossover trial	Nile tilapia—*Oreochromis niloticus*	20 g single dose of FPH, WPH dissolved in 100 mL water or 5 g PLA (sucralose; six capsules) taken with 100 mL water	N/A	*Primary outcome:* FMD at 10, 30, 60, and 120 min postprandial. *Secondary outcome*: Total antioxidant capacity (TAC) of each condition.	Endothelium-dependent dilation increased at 30 min following WPH but not FPH. TAC was greater in the FPH compared with the WPH and PLA.
Siegler et al. [77]	Healthy (median (IQR): age 23 (6) years), aerobically trained males (mean ± SD: VO_2_max 52.5 ± 5.2 mL·kg^−1^·min^−1^)	Randomised, double-blind, crossover design	Salmon	180 mL of the following beverages every 15 min during exercise (CHO only: 67 g·h^−1^ maltodextrin; CHO-PRO: 53.1 g·h^−1^ maltodextrin and 13.6 g·h^−1^ WPC; CHO-PRO-PEP: 53.1 g·h^−1^ maltodextrin, 13.6 g·h^−1^ WPC, and 2.4 g·h^−1^ FPH)	90 min bout of cycling at 50% W_max_ followed by a 5-km time trial	*Primary outcome:* 5-km TT time to completion, power output. *Secondary outcomes*: RER, HR	Mean 5-km TT time to completion and power output did not differ between trials. RER in CHO-PRO was higher than CHO and CHO-PRO-PEP.
Vegge, Rønnestad and Ellefsen [78]	Well-trained male cyclists (age 22 ± 2 years; VO_2_max 65 ± 4 mL·kg^−1^·min^−1^)	Randomised, double-blind, crossover trial	Nutripeptin^TM^ (Np; codfish-based)	Either a CHO beverage containing 8.3% maltodextrin (60 g·h^−1^), PROCHO (maltodextrin and 2.1% intact whey protein, 12.4 g·h^−1^), or NpPROCHO (maltodextrin and whey plus 0.4% Np (2.7 g·h^−1^).	Prolonged cycling (120 min) at 50% W_max_ followed by 5 min mean-power test	*Primary outcome*: 5-min mean-power performance. *Secondary outcomes*: HR, VO_2_, RER, RPE, blood glucose and lactate, blood urea nitrogen	5-min mean-power did not differ between beverages. There were no differences between beverages for HR, RER, VO_2_, glucose, lactate, or RPE. Blood urea nitrogen significantly increased from baseline to 120 min in NpPROCHO and PROCHO only.
Zaïr et al. [79]	Healthy, overweight (BMI: 25–30 kg/m^2^) women (age 18–50 years)	Randomised, double-blind, placebo-controlled crossover trial	Blue whiting	1 g FPH or placebo (microcrystalline cellulose) twice daily for two weeks, followed by a two-week washout period. Testing sessions lasted 420 min, with products consumed at 0 min and 240 min, followed by an ad libitum lunch at 270 min	N/A	*Primary outcome*: Subjective appetite sensations. *Secondary outcomes*: Plasma glucose, insulin, CCK, and GLP-1. Ad libitum energy intake	No differences in hunger AUC between FPH and placebo. Lower plasma glucose at 270 min in FPH compared with PLA. No differences in plasma insulin, CCK and GLP-1 or ad libitum energy intake

Abbreviations: AUC, area under the curve; BCAA, branched-chain amino acid; BMI, body mass index; CCK, cholecystokinin; CHO, carbohydrate; CRP, C-reactive protein; EAA, essential amino acid; FMD, flow-mediated dilation; FPH/PEP, fish protein hydrolysate; GLP-1, glucagon-like peptide 1; HR, heart rate; IBS-SSS, Irritable Bowel Syndrome Severity Scoring System; IQR, interquartile range; MCP-1, monocyte chemoattractant protein 1; N/A, not applicable; PLA, placebo; PRO, protein; QoL, quality of life; RER, respiratory exchange ratio; RPE, rating of perceived exertion; SCFA, short-chain fatty acid; SD, standard deviation; StO2, skeletal muscle oxygen saturation; TAA, total amino acid; TAC, total antioxidant capacity; TT, time trial; TTE, time to exhaustion; WPC, whey protein concentrate; WPH, whey protein hydrolysate; VO_2_, volume of oxygen; VO_2_max, maximal oxygen uptake.

**Table 2:** Essential amino acid composition of various FPH alongside their comparative WPH from among the reviewed studies. Approach adapted from van Vliet et al. [97].

Author(s)	Source Species	Essential Amino Acids, mg/g	Leucine, mg/g
FPH			
Alvares et al. [59], Oliveira et al. [60]	Nile tilapia	772.01 ± 107.17	75.07 ± 14.24
Cordeiro et al. [68]	Nile tilapia	347.40 ± 48.23	33.78 ± 6.41
Dale et al. [69,70,71], Jensen et al. [73]	Atlantic cod	294.3	60.3
Drotningsvik et al. [72] *	Blue whiting	375.3	72.7
WPH			
Cordeiro et al. [68]	Not specified	458.60 ± 42.20	49.43 ± 5.74
Oliveira et al. [60]	Not specified	1019.05 ± 93.79	109.85 ± 12.76

Notes: FPH, fish protein hydrolysate; WPH, whey protein hydrolysate. The asterisk indicates that EAA content was calculated in the absence of exact mg/g measures using information provided in the article.
